# Supplementary material for: Transcriptomic Analysis of TDP1-Knockout HEK293A Cells Treated with the TDP1 Inhibitor (Usnic Acid Derivative)
Source: Int J Mol Sci. 2025 Sep 23;26(19):9291. doi: 10.3390/ijms26199291 (PMC12525351; doi:10.3390/ijms26199291)
Supplement: Supplementary file 1 [file ijms-26-09291-s001.zip › Supplement DEGs in HEK392A Tdp1_090925.pdf]

Transcriptomic Analysis of TDP1-Knockout HEK293A Cells Treated with the TDP1 Inhibitor (Usnic Acid Derivative)

Alexandra L. Zakharenko [1], Nadezhda S. Dyrkheeva [1], Andrey V. Markov [1], Maxim A. Kleshchev [1,2], Elena I. Ryabchikova [1], Anastasia A. Malakhova [1,2], Konstantin E. Orishchenko [2], Larisa S. Okorokova [3], Dmitriy N. Shtokalo [3,4], Sergey P. Medvedev [1,2], Suren M. Zakian [1,2], Alexey A. Tupikin [1], Marsel R. Kabilov [1], Olga A. Luzina [5], Sergey M. Deyev [6] and Olga I. Lavrik [1,7,*]

1 Institute of Chemical Biology and Fundamental Medicine, Siberian Branch of the Russian Academy of Sciences, 8 Lavrentyeva Ave., 630090 Novosibirsk, Russia; a.zakharenko73@gmail.com (A.L.Z.); dyrkheeva.n.s@gmail.com (N.S.D.); andmrkv@gmail.com (A.V.M.); max82cll@ngs.ru (M.A.K.); lenryab@niboch.nsc.ru (E.I.R.); amal@bionet.nsc.ru (A.A.M.); medvedev@bionet.nsc.ru (S.P.M.); zakian@bionet.nsc.ru (S.M.Z.); alenare@niboch.nsc.ru (A.A.T.); kabilov@niboch.nsc.ru (M.R.K.)

2 Federal Research Center Institute of Cytology and Genetics, Siberian Branch of the Russian Academy of Sciences, 10 Lavrentyeva Ave., 630090 Novosibirsk, Russia

3 AcademGene LLC, 6 Lavrentyeva Ave., 630090 Novosibirsk, Russia; larisaok123@gmail.com (L.S.O.); dmitry@novel-soft.com (D.N.S.)

4 A.P. Ershov Institute of Informatics Systems Siberian Branch of the Russian Academy of Sciences, 6 Lavrentyeva Ave., 630090 Novosibirsk, Russia

5 N. N. Vorozhtsov Novosibirsk Institute of Organic Chemistry, Siberian Branch of the Russian Academy of Sciences, 9 Akademika Lavrentieva Ave., 630090 Novosibirsk, Russia; luzina@nioch.nsc.ru

6 Shemyakin-Ovchinnikov Institute of Bioorganic Chemistry, Russian Academy of Sciences, 16/10 Miklukho-Maklaya Str., 117997 Moscow, Russia; deyev@ibch.ru

7 Department of Physical and Chemical Biology and Biotechnology, Altai State University, Pr. Lenina 61, 656049 Barnaul, Russia

* Correspondence: lavrik@niboch.nsc.ru

Supplementary Materials

Table S2. Differentially expressed genes forming OL9-119-sensitive regulome.

Gene	Degree *	Log_2(Fold Change)	Gene	Degree *	Log_2(Fold Change)
HSP90AA1	35	-0.608	SNCA	13	-0.778
ACTB	33	-0.599	MMP9	13	2.272
JUN	30	0.685	H2AC8	13	5.561
NDUFAB1	28	-0.607	H2AC6	13	1.265
COX5A	27	-0.594	LSM3	13	-0.916
EGFR	26	0.7	SIRT1	12	0.599
UQCRQ	24	-0.831	XBP1	11	0.685
HSPA8	23	-1.814	CENPA	11	-0.767
ATP5MF	22	-0.646	DNAJB1	11	-1.112
NDUFA4	21	-0.75	TXN	11	-0.613
SNRPE	21	-0.999	PPIH	11	-0.743
ATP5ME	21	-0.777	ASNS	11	1.598
NDUFA12	21	-0.77	IL1A	11	2.767
SNRPD2	21	-0.707	NEDD8	11	-0.734
SNRPG	21	-0.808	ATP5MC2	11	-0.63
H4C14	20	2.642	HSPA1B	11	-2.42
SNRPD1	20	-0.755	ICAM1	11	0.849
UQCR11	20	-0.866	CALM2	11	-0.642
ATP5PF	20	-1.081	CEBPB	11	2.146
POLR2L	20	-0.866	MRPL33	10	-1.167
SNRPD3	20	-0.645	HSPE1	10	-1.122
SNRPF	19	-0.815	ZCRB1	10	-0.636
NDUFA6	19	-0.659	SNRPC	10	-0.62
NDUFS5	19	-0.777	H3C6	10	-1.937
COX7A2	19	-0.859	CRIPT	10	-0.679
NDUFA2	18	-0.88	SNRNP25	10	-0.601
RBX1	18	-0.648	SDHD	10	-0.613
NDUFS6	18	-0.728	RPL36AL	9	-0.738
NDUFB4	18	-0.975	SEC61B	9	-0.747
NDUFB3	18	-1.072	DNAJA1	9	-0.962
SF3B5	18	-0.864	SLC3A2	9	0.62
CYCS	17	-0.657	SUMO1	9	-0.654
BCL2	17	0.939	MMP2	9	-0.642
ATP5MD	16	-0.775	ABL1	9	0.653
COX7B	16	-1.01	CACNA1F	9	-6.597
NFKBIA	16	0.73	PRPF18	9	-1.2
PHF5A	15	-0.781	PRDX1	9	-0.964
CWC15	15	-0.618	MRPL54	9	-0.776
NDUFA3	15	-0.761	SEC61G	9	-1.063
POLR2K	15	-0.903	DDIT3	9	1.851
SF3B6	15	-0.686	MRPL51	9	-0.676
ATP5MC3	14	-0.8	H2BC4	9	2.512
ATP5MC1	14	-0.919	UBE2N	9	-0.586
ATF3	14	1.161	PARK7	9	-0.639
MRPL13	14	-0.824	ISY1	9	-0.684
RPS26	13	-0.668	CFL1	9	-0.587

Gene	Degree *	Log$_2$(Fold Change)
TRIB3	8	1.553
DNAJB4	8	-0.916
GTF2A2	8	-0.61
MED9	8	-0.593
GTF2H5	8	-1.293
JAK2	8	0.631
BCL6	8	0.915
MRPL40	8	-0.627
TIMP1	8	-0.662
MRPL23	8	-1.016
MED21	8	-0.631
MED11	8	-0.67
HSPA1L	8	4.667
PTTG1	8	-0.781
HSPH1	8	-0.598
PPIA	8	-0.84
FANCE	7	0.845
ARNTL	7	0.695
SLC7A5	7	1.227
WDR61	7	-0.625
PER2	7	0.967
ATP5MPL	7	-1.026
AURKA	7	-0.602
MRPL48	7	-0.692
OST4	6	-0.713
OSTC	6	-0.707
TNFAIP3	6	0.86
CENPW	6	-1.083
CKS2	6	-0.599
SUPT4H1	6	-0.592
PPP1R15A	6	0.735
MUC1	6	-2.829
CAMK2A	6	5.094
JUNB	6	0.781
MYL6	6	-0.837
CENPX	6	-0.834
DAD1	6	-0.801
TERT	6	3.053
DDX28	6	0.818
SUMO2	6	-0.681
CEBPG	6	1.118
GEMIN7	6	-0.81
CHCHD4	6	0.776
TIMM17A	5	-0.767
PER1	5	0.723
CDKN3	5	-0.81
PRKG2	5	1.862
BHLHE40	5	0.81
FKBP1A	5	-0.586
STAT4	5	3.357
CACNB4	5	1.365
S100A10	5	-0.949
NR1D1	5	1.118
SFN	5	-0.764
ENY2	5	-0.648
TCERG1L	5	-2.603
RPS27L	5	-0.782
MYL9	5	-0.864
SRP9	5	-0.741
TRAPPC3	5	-0.62
TRAPPC10	5	0.609
SSR2	5	-0.837
MMP7	5	-4.518
CACNA1H	5	0.626
ATP6V0E1	5	-0.78
CXCL2	5	2.383
PSAT1	5	1.074
CDC26	5	-0.722
LUM	4	-2.204
NR1D2	4	0.662
ATP6V0A4	4	-4.902
KCNH2	4	-0.889
ENO4	4	3.288
NTS	4	-2.84
SMKR1	4	3.345
COX14	4	-1.178
GABARAPL2	4	-0.818
JDP2	4	1.468
MYLK	4	0.666
DPY30	4	-0.757
H1-0	4	0.654
SLC7A1	4	1.093
CDK5	4	-0.646
KRTCAP2	4	-0.97
CACNB1	4	1.037
FOSB	4	1.069
NRGN	4	-0.702
CACNA1E	4	4.879
SELE	4	4.531
SGF29	4	0.616
DCTN3	4	-0.673
LOC107987373	4	-1.016
PSPH	4	1.174
UTP3	4	-0.714
TRAPPC2L	4	-0.861

Gene	Degree *	Log2(Fold Change)
NEDD4	4	0.897
GADD45B	4	0.953
TOMM22	3	-0.604
TUBB3	3	4.215
SH3GL2	3	0.644
GLI1	3	1.583
PCLAF	3	-0.636
SLC7A11	3	2.748
KLF4	3	0.657
PSMB5	3	-0.621
CHAC1	3	5.008
TAC1	3	1.03
COX17	3	-0.63
CST3	3	-0.591
BNIP1	3	0.595
MED13L	3	0.74
GRPEL2	3	0.694
KCNQ4	3	1.454
COA3	3	-1.387
TRAPPC2B	3	-1.548
TMA7	3	-0.696
CGAS	3	3.7
CCNB3	3	1.293
CDKN2B	3	0.704
ALDOC	3	-1.093
VAMP8	3	-0.831
MTHFD2	3	0.966
GAB2	3	1.143
ATOX1	3	-1.281
ANAPC15	3	-0.746
UBE2L3	3	-0.586
FBXW8	3	0.741
CXCL3	3	2.091
CEBPD	3	1.396
TRAPPC2	3	0.623
LCP1	3	1.128
TXNIP	3	-1.012
GRP	3	4.587
GARS1	3	0.636
TFRC	3	0.931
SLC1A2	3	1.284
FEN1	3	-0.626
RNF135	3	2.41
STX8	3	-0.825
MAFB	3	0.682
MMP10	3	-0.685
CSTB	3	-0.808
ALOX15	3	3.614

Gene	Degree *	Log2(Fold Change)
PCK2	3	1.655
TAGLN2	3	-0.592
NRIP1	3	1.076
MITF	3	0.899
PFDN5	3	-0.694
IKBKE	3	-0.98
VAV3	3	-0.841
AARS1	3	0.731
S100A13	2	-1.215
HLA-DQB1	2	-0.725
RAB5C	2	-0.606
IDI1	2	-0.595
MYLK2	2	-2.592
TNFSF9	2	-0.765
DYNLL1	2	-1.219
FBXO43	2	5.015
LYRM4	2	-0.607
HIGD1A	2	-0.885
IL4I1	2	2.237
ITGA9	2	1.779
TXNDC17	2	-0.65
SFPQ	2	-0.717
DNAJC12	2	-0.593
JAG1	2	0.771
TCHH	2	-1.407
PHC1	2	0.687
MMP12	2	-0.732
MLKL	2	-0.765
TRAF3IP2	2	0.723
RELB	2	0.783
LRRK1	2	0.638
CHMP2A	2	-0.594
TUBB2B	2	0.65
RFXANK	2	-0.689
TP53INP1	2	-0.682
SMPD3	2	2.484
PGAM1	2	-0.741
BBC3	2	0.683
GLRX	2	-0.865
CTH	2	2.05
NEUROG2	2	2.764
ACTC1	2	4.535
DEGS1	2	-0.756
COL18A1	2	
SLC1A4	2	0.75
RNF43	2	-0.594
PSME2	2	-0.698
SLC38A2	2	0.823

Gene	Degree *	Log2(Fold Change)
TRPV6	2	1.078
DCTN6	2	-0.67
POLE4	2	-0.594
PDXP	2	1.684
SDS	2	
AOX1	2	-2.137
GCC1	2	0.606
NLRC5	2	1.014
MLH3	2	1.285
SGPP2	2	0.637
CCNO	2	1.071
UXT	2	-0.805
COL14A1	2	0.767
USP25	2	0.895
CARS1	2	1.006
HLA-DRB1	2	-0.997
CMC2	2	-0.663
POLR3GL	2	-0.86
ZSCAN20	2	1.212
AFMID	2	-0.682
KSR1	2	0.674
PFDN6	2	-0.882
GPT2	2	0.902
DAPK2	2	0.822
KPNA2	2	-0.671
DCUN1D2	2	0.811
SLC43A1	2	0.716
FUNDC1	2	-0.645
PGPEP1	2	0.723
LOC102724159	2	1.933
MYO1F	2	3.741
RAD50	2	1.032
LY96	2	-4.658
SH3BP2	2	0.644
WDR83	2	0.716
TNFRSF9	2	0.712
ALOX5	2	0.952
SESN2	2	1.056
CHML	2	0.707
ITGB8	2	-0.713
PDZD11	2	-0.643
C1GALT1C1	2	-1.191
L1CAM	2	0.631
PROCR	2	-0.716
HLA-DMB	2	-1.075
GTF2IRD1	2	0.776
ARHGEF2	2	0.892

Gene	Degree *	Log2(Fold Change)
SYT9	1	0.666
ST3GAL1	1	0.631
HHIP	1	2.098
MCIDAS	1	0.981
GAP43	1	-1.15
PRDX4	1	-0.723
IRAK3	1	4.084
ALAS1	1	0.788
NANP	1	0.766
EFNA3	1	-1.236
HECA	1	0.592
NEURL1B	1	-0.63
RNF207	1	0.973
KANSL2	1	0.694
GLRX2	1	0.835
PRICKLE3	1	0.789
PIWIL4	1	2.191
SNAPC1	1	0.742
FLG	1	3.982
EEF2K	1	0.696
NOS1AP	1	1.168
THYN1	1	-0.761
DDIT4	1	1.912
HRK	1	0.744
S100A11	1	-0.745
SLC7A9	1	-3.317
VIPAS39	1	0.651
FBXL7	1	4.274
MCTS1	1	-0.874
SH3BGRL	1	-0.672
FBXO4	1	0.77
HK2	1	0.63
EXTL2	1	-0.639
KCNJ4	1	1.459
IQCJ-SCHIP1	1	1.081
RAB43	1	3.201
FKBP1B	1	-1.04
LGALS1	1	-0.651
CHFR	1	0.797
RHOH	1	3.577
TTC4	1	-2.313
ATXN7L1	1	1.29
CSTF3	1	-0.675
CDO1	1	-0.669
INO80D	1	0.678
HERPUD1	1	1.065
CETN3	1	-0.847
PLIN1	1	3.067

Gene	Degree *	Log_2(Fold Change)
KIF27	1	0.888
LTC4S	1	2.794
WDR83OS	1	-0.856
MYBPC2	1	4.3
PIN4	1	-0.745
TIMM23B	1	0.787
MT2A	1	-0.868
CLIP2	1	0.687
ALDH1L2	1	1.888
ZBED9	1	0.629
ALDH1A2	1	0.63
TMEM126A	1	-0.636
CDKAL1	1	0.588
UNC5B	1	0.791
MUTYH	1	1.198
SUGT1	1	-0.586
APOO	1	-0.691
SH2B3	1	0.969
NOTCH4	1	-1.849
PAGR1	1	1.679
MAGIX	1	-1.747
BACH1	1	0.617
DNM3	1	1.069
ENTPD2	1	-1.568
RASGEF1B	1	0.758
CAMK1G	1	1.78
PAX3	1	0.754
POMP	1	-0.984
KCNMB3	1	1.288
ARRDC3	1	1.668
PIP5K1B	1	2.096
MSH4	1	2.974
DCP2	1	-0.645
MYCL	1	0.896
USP4	1	0.722
TRIM66	1	0.937
NUPR1	1	1.091
ARHGAP31	1	0.788
SCHIP1	1	1.081
GCA	1	-0.604
ISL2	1	0.846
PHLDA1	1	0.702
RSPO4	1	4.499
SNCB	1	2.036
RFX5	1	0.612
SERPING1	1	-0.973
UCHL3	1	1.207
DPF3	1	2.073

Gene	Degree *	Log_2(Fold Change)
IFRD1	1	0.703
ZNF213	1	0.787
MTSS1	1	1.184
CD14	1	2.542
AGTR1	1	-4.316
ERG28	1	-0.749
IL20RB	1	-2.376
OPN3	1	1.85
PLA2G4D	1	5.207
HTR2C	1	5.266
PAGE4	1	-3.191
TEC	1	0.941
MMP24	1	2.273
SH2B2	1	1.514
TIRAP	1	0.651
MLNR	1	2.868
CNKSR2	1	0.87
GRIA4	1	-5.017
ETFB	1	-0.72
SULT1C4	1	2.106
DLX2	1	0.891
EIF2S3B	1	0.84
ME3	1	-1.344
GDF15	1	1.923
PELI1	1	0.781
CISD2	1	-0.612
CTC1	1	0.604
CMC1	1	-0.72

* Degree means the connectivity level, the number of connections with neighbors in the network
